# Implementing Baby Friendly Hospital Initiative policy: the case of New Zealand public hospitals

**DOI:** 10.1186/1746-4358-2-8

**Published:** 2007-04-23

**Authors:** Trinie Moore, Robin Gauld, Sheila Williams

**Affiliations:** 1 Plunket Society, PO Box 1275, Dunedin, New Zealand; 2 Department of Preventive and Social Medicine, University of Otago, PO Box 913, Dunedin, New Zealand

## Abstract

**Background:**

Studies show that when the Baby Friendly Hospital Initiative (BFHI) is implemented breastfeeding rates increase. However, there are likely to be various barriers to BFHI implementation. This article reports on an empirical study of government-directed BFHI implementation in the New Zealand public hospital system. It focuses primarily on the barriers encountered through implementing the first Two Steps of the BFHI: developing BFHI policy and communicating it to staff; and providing necessary staff training.

**Methods:**

Qualitative interview data were collected from six lactation consultants. These interviewees emerged via a purposive sample of public hospitals that represent the full range of New Zealand public hospitals. Using a content analysis technique, key themes were drawn from the transcribed interview data.

**Results:**

Analysis revealed eight themes: the hospitals were in varying stages of BFHI policy development; hospital policy was not necessarily based on government policy; hospital policies were communicated in differing ways and dependent on resources; factors outside of hospital control impacted on capacity to improve breastfeeding rates; and complex organisational matters pose a barrier to educating personnel involved in the birthing process.

**Conclusion:**

The findings of this study provide empirical support for prior articles about the process of BFHI policy development and implementation. The study also shows that implementation is multi-faceted and complex.

## Background

Breastfeeding is the normal method of infant feeding, but many people choose to feed their newborn babies on infant formula. While babies grow and develop with either method of feeding, there is evidence of disadvantages for infants that are not breastfed [1-3], and for their mothers [4-6]. Some studies have indicated that breastfeeding has economic, social and environmental benefits [7]. These benefits have been recognised by the World Health Organization (WHO) and UNICEF who jointly launched a new international initiative aimed at protecting, promoting and supporting breastfeeding in 1991 [8]. Titled the 'Baby Friendly Hospital Initiative' (BFHI), this is intended to give every baby the best start in life by ensuring that, in environments such as hospital birthing units, breastfeeding is promoted as the norm and not supplemented by infant formula. The BFHI policy statement included Ten Steps to guide every maternity service in the quest to become 'baby friendly' (see Table 1).

**Table 1:** Ten Steps to successful breastfeeding*

Every facility providing maternity services and care for newborn infants should:
1. Have a written breastfeeding policy that is routinely communicated to all health care staff.
2. Train all health care staff in skills necessary to implement this policy.
3. Inform all pregnant women about the benefits of breastfeeding.
4. Help mothers initiate breastfeeding within one hour of birth.
5. Show mothers how to breastfeed and how to maintain lactation, even if they should be separated from their infants.
6. Give newborn infants no food or drink other than breastmilk, unless medically indicated.
7. Practice rooming-in – allow mothers and infants to remain together 24 hours a day.
8. Encourage breastfeeding on demand.
9. Give no artificial teats or pacifiers to breastfeeding infants.
10. Foster the establishment of breastfeeding support groups and refer mothers to them on discharge from the hospital or clinic.

*World Health Organization: **Protecting, Promoting and Supporting Breastfeeding: The Special Role of Maternity services**. Geneva: World Health Organization; 1989.

To date, there have been a number of published studies of the impact of the BFHI and of initiatives to increase breastfeeding in line with BFHI principles. For example, there is evidence that implementing the Ten BFHI Steps will increase breastfeeding rates and duration [9,10], although one study found that the presence of formula and pacifiers was not associated with lower frequency or shorter duration of breastfeeding [11]. There is also evidence that staff often require training in order to change their practices to promote the Ten BFHI Steps [eg [12-14]]. One study found that specific staff training in the BFHI was an important component in obtaining their compliance with and commitment to implementing the Ten Steps [15]. This training was also found to significantly increase exclusive breastfeeding rates on hospital discharge. Studies have also found that mothers assigned to a BFHI intervention had increased rates of breastfeeding, especially for exclusive breastfeeding [16,17].

There have also been reports of how hospitals and health services have reorganised to become 'baby friendly'. These include commentaries on the managerial process of changing staff practices to implement the Ten Steps [18], and on the requirement for multi-disciplinary approaches that involve the various health professionals and service providers who come into contact with new mothers and their babies [19]. A Turkish study found that in none of five hospitals analysed were all Ten Steps implemented [20]; that staff were often too busy or under-resourced. Meanwhile, a report from Sweden provided information on how the BFHI was implemented across all public hospitals and birthing centres and discussed some of the barriers encountered there and in other countries [21].

There are no specific reports from the perspective of maternity service providers of the processes involved and impediments when a government decides that the BFHI ought to be implemented in all public hospitals as happened in New Zealand in 2001. This article, therefore, reports on a New Zealand study carried out in 2004 that sought to explore the processes and challenges of implementing national policy at the hospital level. Its primary focus is on Steps One and Two of the BFHI which are respectively aimed at developing and communicating breastfeeding policy to staff, and providing the necessary training for staff in order to be able to implement the BFHI.

The New Zealand case is an interesting one for several reasons. First, New Zealand has a 'national' health system in which there is universal access, free of patient charges, to hospital and maternity services. The New Zealand government funds around 80% of all health care and the costs of most births are fully state funded.

Second, the way maternity services are funded and regulated means that a high proportion of birthing and perinatal care is provided by 'independent' midwives. These practitioners operate as private business people, from their own clinics, but are funded by the government per birth case. However, they routinely use the facilities of public hospitals for the actual birth. Public hospital birthing services also directly employ their own midwives, nursing and medical staff including obstetricians. Expectant mothers can opt for an independent or hospital midwife at the beginning of their pregnancy.

Third, the planning and organisation of publicly-funded health services is devolved to regional governing boards. Thus, the government provides funding and policy guidelines, but when it comes to implementation there are considerable differences at the local hospital level. In terms of Steps One and Two of the BFHI, the regional governing boards are required in their funding contract with the government to ensure that each public hospital, which the regional governing board holds a separate contract with, has both a policy and training programme for staff. In turn, in order to comply with their funding contracts, the hospitals must demonstrate, at least on paper, that they are developing or have the mechanisms in place to implement Steps One and Two.

Fourth, the government was a relatively late adopter of the BFHI. Through the 1990s there had been an emerging consensus within the government's chief policy agency, the Ministry of Health, and across the maternity sector, that breastfeeding was best, and recommendations to this effect for the sector had been produced [22]. It was not until 2001 that the government became the 133^rd ^in the world to formally launch the BFHI initiative and develop a hospital accreditation programme [23]. In 2002 the first hospital achieved accreditation and others have subsequently followed. Since the BFHI adoption, the government has moved to further promote breastfeeding by establishing a series of dates by which it expects rates to have improved [24]. These include increasing, by 2010, exclusive breastfeeding at six weeks to 90% and at three months to 70%. New provisions for paid parental leave of up to 14 weeks were also introduced in 2002, with the potential to provide for circumstances that further support breastfeeding [24].

## Methods

New Zealand's public hospital services are divided into 21 regional groups. Most provide services across multiple locations, all provide at least secondary services and some feature both secondary and tertiary service providers. The hospital groups serve differing populations and there are considerable variations in socio-economic and ethnic composition, and also in terms of urban concentration and geographical coverage. To capture these dynamics, we purposively sampled six hospital organisations for analysis. The sample included one very large urban tertiary hospital that serves over 1 million people (a quarter of New Zealand's population). This population has over-representation of ethnic minority and lower socio-economic groups. We also sampled a tertiary hospital that serves a half million people and which has an above average representation of New Zealand's white population. We sampled two secondary hospitals serving populations of approximately one hundred thousand people. One had a higher than average proportion of indigenous Maori; the other of white people. Finally, we selected two small rural hospitals serving populations of less than fifty thousand. Again, one had a higher than average Maori population, the other of whites.

Ethical approval for the study was granted from the University of Otago Ethics Committee, after which a senior lactation consultant (LC) from each of the six hospitals was contacted with a request for interview. All agreed to take part. The six were then sent an information sheet, consent form and a list of subject areas that formed the basis of the interviews. The interviews, conducted during August-September 2004 by telephone and tape recorded, covered:

• Whether their hospital organisation had a breastfeeding policy;

• How this related to the government's policy statement entitled "Breastfeeding: A Guide to Action" [24];

• How the policy was communicated to staff within the organization;

• What barriers there were to implementation of the policy;

• How the policy was evaluated;

• Overall, how effective the policy was perceived to be.

Interview tapes were transcribed and a content analysis undertaken on the text where we sought to identify, code and categorise the themes that emerged from the data [25,26]. Some of the themes were consistent across all hospitals whilst others only emerged for one hospital but appeared to be important. The next section describes and illustrates, with interview extracts, the main themes that emerged.

## Results

### Theme one: Policy development

We found that, three years on from the New Zealand government's policy statement, all six of the hospital organisations had developed a breastfeeding policy, although there were variations in the extent to which these were finalised and in circulation as commented on by interviewees:

*"We, our policy, our breastfeeding policy is currently still in draft form." *(Small hospital A)

*"Well, we have a breastfeeding policy that certainly includes the words promote, protect, you know the same ones as the WHO . . . So, our policy statement says, that all . . . staff have a role in implementing the Breastfeeding Policy and will be trained in the skills necessary to implement it." *(Medium hospital A)

The foundation of the various breastfeeding policies appeared to have been "The Baby Friendly Hospital Initiative". However, the BFHI only reached the hospital policy agenda due to the 2001 directive from the New Zealand Ministry of Health. The following illustrates this:

*". . . but fortunately because the Ministry of Health is actually breathing that directive, you know that BFHI is now appearing at much higher management, you know what I mean, so up higher than my line manager." *(Medium hospital A)

### Theme two: Relationship between hospital and government policy

On the whole the six interviewees felt that, while the principles of their policy were similar to the government's policy statement "Breastfeeding: A Guide to Action", the government's policy was too abstract: it provided a series of principles but was short on practical detail of direct relevance to hospital-level implementation:

*". . . the WHO documents, as adapted for New Zealand, is actually where we took the policy from because our policy, by the very nature, has to go to the World Health Organisation standards and the [New Zealand government] Guide to Breastfeeding sort of, is probably a bit more wishy washy." *(Small hospital B)

*". . . we didn't specifically use [government policy] as a reference. I am aware of the document. We very much work from you know the BFHI Aotearoa documents [based on WHO material]." *(Large hospital A)

However, one respondent felt that their policy was closely related to "Breastfeeding: A Guide to Action" and was able to state how it related to all the action plan steps:

*"It really meets all their goals I guess. Step One is a Baby Friendly Hospital, is a goal of the action plan, Maori and Pacific Island input and their awareness, it's the audit of the process and we've got to state, it's the breastfeeding promotion by consultation, we cover the antenatal education and it's to increase breastfeeding rates, support the universal definitions and stuff like that . . . and to encourage good postpartum care. So it's all those things" *(Large hospital B)

### Theme three: Communicating policy

Respondents outlined a number of ways that they communicated their policies to staff, including posters on the walls of hospitals wards, on the internet and intranet, staff group education and through one-on-one education sessions:

*". . . it's up on the wall in a poster format . . . also in every room in our postnatal areas and it's in every, well it's in every area actually." *(Medium hospital B)

Respondents noted several issues that made communicating the policy to hospital midwifery and nursing staff difficult. The first of these was where the policy was still in draft form it was often not accessible by staff:

*". . . it is rather hard when the system is so slow getting it from being written to getting where the staff can access it . . . to give them something that says the second draft it's not actually quite the same as saying here is the policy." *(Medium hospital A)

At some hospitals it was difficult to ensure that the policy was communicated to all staff. Frequent nursing and midwifery staff changes meant that there was always at least one new member of the team who had not had any education relating to the policy. Whilst they might have been shown the location of the policy when they first entered the ward, it was difficult for them to have time to take the information on board, as the workload was large:

*"We've got nearly 200 nursing and midwifery staff and when I look at our database on education, we've also got a database on those who have left the hospital so those have already been trained and left the hospital are almost as great as those who are still here. So no matter how much training you do, there's always new staff coming." *(Large hospital A)

At some hospitals there was also limited education time for staff to learn about the breastfeeding policy. It was expected that nurses and midwives would receive 18 hours of breastfeeding education in order for the facility to attain Baby Friendly Hospital accreditation. However, there were often competing needs for the education of staff in tertiary hospitals, meaning that breastfeeding policy education was assigned a lower priority. This was because the hospitals had a higher proportion of complicated deliveries and sick mothers so the staff needed to be skilled in ensuring that these mothers were safely looked after – and to achieve this skill, specific education was required:

*". . . staff have to be on board with pain relief, they have to be on board with IV fluids and any new staff are just coping with all sorts of things . . . " *(Large hospital A)

Another difficulty was a lack of resources for educational purposes or policy development, as New Zealand public hospitals are required to work within an allocated government funding level:

*"We're still doing staff education but there's been a bit of a halt to that because of you know financial restrictions." *(Medium hospital A)

*"This one [policy] is still in second draft . . . but the person who is actually doing this is our quality manager and the typist who's been working so hard and they've both just been made redundant." *(Small hospital B)

There was also difficulty ensuring that the policy was relayed to casual nursing and midwifery staff. When agency nurses or staff from other wards were required, there was not the time to ensure that they were familiar with the policy and the implications of it for working in the area:

*"And so we are trying to get some of the bureau staff that come to the hospital to come to the breastfeeding study day . . . there are certain ones from the bureau that they won't take but they get desperate and will take anybody at times as there never seems to be enough staff around." *(Large hospital A)

An international standard for Baby Friendly Hospital Accreditation is that all non-nursing staff also receive three hours a year breastfeeding education. This is so all staff, other than nurses and midwives working in hospitals, are promoting breastfeeding. This also caused difficulties with some staff as they did not understand why they needed such education and they were reluctant to participate. One respondent commented that communicating policy to the staff was only one step in the process. Getting them to change their practice to comply with policy took time. This was the case in a small rural hospital, which did not have other back-up services on the premises:

*"I see it as there is a certain reluctance to go away from what is known . . . having that in mind because we haven't got a paediatrician next door that we can yell out to if something goes wrong . . . at the moment some of the staff perceive [the BFHI] as a risk, which it might be." *(Small hospital A)

A couple of respondents also discussed the problems with restructuring hospital services within their area (which is ongoing in the New Zealand health sector) and the impact this would have on implementation:

*"It's very difficult when you're about to move hospitals . . . but there's been quite a lot of people who didn't know whether they wanted to move with the hospital and people have left and while people were thinking about leaving, they weren't interested in education." *(Large hospital B)

### Theme four: Overcoming barriers to communicating policy

All interviewees discussed barriers to communicating the BFHI policy. However, the respondent from the hospital that had achieved accreditation felt that getting the policy to a point where all the staff had received their 18 hours education and having management support made a huge difference:

*"And of course it's because we've had the policy up and running and we've done all the consultation and you know it was one of the biggest things, and also education because I had been employed here really. Not anything to do with me. It was just the fact that there was a lactation consultant position here so therefore that was the function of the LC is to educate so that the education hours had been attained which is one of the biggest things . . . " *(Large hospital B)

A stable workforce also made a difference to implementing the policy:

*". . . like I've been here for 30 years and there's a lot of us that have been around for a very long time because of the camaraderie of the place really so you know because of that I suppose we seem to have a good strategy going." *(Large hospital B)

### Theme five: Difficulty achieving exclusive breastfeeding targets

Most interviewees stated that the government's targets for exclusive breastfeeding at discharge would be difficult to reach for the following reasons:

1. Women with health problems and pregnancy complications were referred to hospitals for antenatal, birth and postnatal care. This was particularly the case with tertiary hospitals, where they receive complicated cases from around their regional areas:

*"I mean one of our wards, our high risk ward . . . had 24 percent exclusive [breastfeeding] yesterday and that's because of the nature of the women that go there" *(Large hospital A)

*". . . how sick some of these patients are and they have luers [intravenous access] in their hands and they can't [breastfeed] and they want to but they're grogged up with drugs . . . sometimes their physical condition is such that there is no sign of any lactogenesis triggering off." *(Large hospital A)

2. Some hospitals had a higher rate of caesarean births than the national average:

*"We have a very high caesarean section rate here – we have one of the highest in the country, well over 30 percent – a lot of those are first time mothers and that creates delayed lactation." *(Large hospital A)

3. Sick babies reduce the likelihood that breastfeeding will be established or exclusive:

*". . . in the case of babies that go to the neonatal unit . . . I think when we have difficulty with the baby separated from the mother with this situation. . . the difficulty of getting expressed breast milk for those babies" *(Medium hospital B)

4. Some hospitals were located in areas where there are a high number of women of lower socio-economic status or an ethnic minority who are less likely to have attended antenatal care or to breastfeed:

*". . . but if most of our good mums are going to [private obstetric facility] we're left with the late bookers . . . what happens when you have an elderly primip [arous woman] who doesn't speak the language who is a late booker?" *(Large hospital A)

### Theme six: Policy evaluation

At the time of interviews, it was too soon to say what the long-term effect of the policy on breastfeeding initiation would be. Once a New Zealand hospital achieves BFHI accreditation it is not audited for another three years (although there may be occasional requests for performance data). However, the respondent from the one accredited Baby Friendly Hospital commented that they undertake regular internal audits to assess their performance:

*"Well, things like audits, audits on breastfeeding stats, audits on skin-to-skin contact. I've done audits on my antenatal breastfeeding classes – those type of things. Its measurements really . . . we have them ongoingly. I do breastfeeding stats every six months." *(Medium hospital A)

Many of the hospitals were also comparing their exclusive breastfeeding rate on discharge with the BFHI recommended rate of 75 percent. What was unclear from the interviews was the correct denominator for this rate. One lactation consultant thought that it included all mothers and babies being discharged whilst another hospital thought that it did not include the babies who required formula for a specific medical reason. It was also unclear whether the rate included mothers and babies who were discharged to another maternity facility (that is, they came to the secondary or tertiary hospital only for delivery and then went to another smaller facility) or whether it only included women who went directly home from the hospital.

### Theme seven: Discussing policy with other providers

Respondents suggested their ability to discuss the policy with other providers such as independent midwives impacted on their capacity to improve breastfeeding rates. Ensuring other providers were aware of the policy was relatively easy to achieve with a small area and fewer independent midwives, whereas in a bigger centre it was difficult to know who all the individual midwives were as well as ensure that they knew what the policy was:

*"We've got a large [number of independent midwives] . . . There's a lot of them and the reality is that it appears by the deafening silence that any comments about the policy from them that they just blithely ignore it and say that's what we do here and they come in and just do their own thing." *(Medium hospital A)

Some regions had a breastfeeding advocacy group which was a combination of health professionals involved with breastfeeding mothers. Where these groups were present, it was felt that the policy could more easily be communicated to the professional groups that were represented on it.

*". . . well, local levels, there is the breastfeeding support group here . . . that's been established by [a public health service] . . . that's been alongside them and they've helped us and they had a booklet that they printed for us. So that's been good interaction." *(Medium hospital B)

### Theme eight: Size matters

Interviewees from smaller hospitals noted that it was probably easier to implement the policy because of a smaller number of staff, a more stable workforce, fewer casual staff being employed and a smaller number of lead maternity carers that impact on the implementation of the policy. The interviewees from the two large hospitals, particularly the largest tertiary hospital, suggested that specific barriers they faced included dealing with a large, transient workforce and high maternity staff turnover. This meant they had to frequently rely on temporary staff and that continuity of training was affected.

## Discussion and conclusion

This study illustrates that in the New Zealand public health system there are many barriers to implementing BFHI policy at the public hospital and perinatal service delivery level. In this sense, the study provides empirical support for prior discussions about the processes of BFHI-driven transformation [18,19,21].

The study also demonstrates that implementation is a multifaceted undertaking that is dependent on a range of factors that may support or hinder the process, as widely acknowledged elsewhere [eg [27,28]]. Implementation across the six participating hospital organisations was a staggered and slow process, stimulated not by the hospitals themselves, but by a central government policy directive. More than three years after the New Zealand government put its weight behind the BFHI, only one of the study hospitals had attained accreditation, which was representative of BFHI accreditation in public hospitals at the time; the other five continued to experience difficulties in developing and implementing their policies. This implies that, if hospitals are not themselves leading a drive to become baby friendly, there is a real need for some kind of external motivation, and that this motivation possibly needs to be matched with the right incentives and resourcing to ensure that the BFHI policy is implemented. This is likely to be particularly the case in health systems that are predominantly government funded and where government has a high level of policy responsibility. If government is to provide policy stimulus then, as respondents in this study suggested, the policy needs to have a practical orientation so that it can be easily actioned by service providers.

Notable in this study was the fact that developing a hospital-level policy was only one part of the equation and that gaining staff compliance with that policy took time. Considerable effort and resourcing was also required to ensure that education was far-reaching, and accessible to all relevant staff. Where practitioners beyond the direct jurisdiction and employment of the hospital are involved in delivering services, as in New Zealand, this poses additional challenges. This study also confirms that policy is contingent on the context within which is it being implemented [29].

As noted by respondents in this study, issues such as the socio-economic status of a community and the relative complexity of presenting cases at hospital birthing centres will impact on the capacity to boost breastfeeding rates. Our respondents' comments under Theme Five in this article imply that specific resources and training may need to be provided for birthing centres that deal with complex cases where exclusive breastfeeding may be less likely to be achieved. New Zealand's public hospitals do their best to facilitate rooming in after a caesarean, and to provide a mother's breast milk for her baby if in neonatal intensive care. However, to consolidate the focus on improving exclusive breastfeeding rates there may be a need for additional special education and language translation services, increased lactation consultant numbers, and close attention to the needs of mothers and maternity staff throughout the birthing process.

A further finding from the study, with corresponding lessons, may be that implementing BFHI policy could be more straightforward in smaller hospital organisations and hospitals with more stable workforces. The existence of interdisciplinary and advocacy groups that span hospitals and community may also assist with policy dissemination and compliance.

This qualitative study has the possible limitation of six participants. However, as noted, these people and the organisations they spoke on behalf of were purposively selected and representative of the range of New Zealand public hospitals and their populations. The interviewees expressed themes that were common to all and, in this sense, we believe they would be germane to the wider public hospital sector in New Zealand. We also consider that, through the interview process, we reached saturation and that extending the number of interviewees would not have yielded further knowledge; it may, however, have provided additional examples. The study may also be limited by its New Zealand focus and fact that the studied hospitals were part of a state-funded health service. The world's health care systems differ from one another in terms of regulation, funding and service organisation [30,31] and so our findings and lessons need to be carefully translated. A further limitation is that the study was aimed largely at exploring barriers to implementing Steps One and Two of the BFHI. What is now required are additional studies of BFHI implementation, in particular analysing barriers to implementing the remaining BFHI Steps, undertaken within differing health and hospital systems.

## Competing interests

The author(s) declare that they have no competing interests.

## Authors' contributions

TM conducted the research reported in this article as part of a Master of Public Health thesis supervised by RG and SW. RG drafted the article which TM and SW provided comments on. All authors read and approved the final accepted draft.
